# Deep Learning Detection of Retinitis Pigmentosa Inheritance Forms through Synthetic Data Expansion of a Rare Disease Dataset

**DOI:** 10.21203/rs.3.rs-8002154/v1

**Published:** 2025-12-02

**Authors:** Elizabeth E. Hwang, Max L. Rivera, Lin Jia, Man Ting Lin, Krish Nachnani, Olivia Yuan, Pulkit Madaan, Ying Han, Jacque L. Duncan, Jing Shan

**Affiliations:** University of California, San Francisco; University of California, San Francisco; Digillect LLC; University of California, San Francisco; University of California, San Francisco; University of California, San Francisco; University of California, San Francisco; University of California, San Francisco; University of California, San Francisco; University of California, San Francisco

## Abstract

Accurate classification of inheritance patterns is an integral part of diagnosis and genetic counseling for inherited retinal diseases (IRDs). Traditionally reliant on pedigree analysis, clinical phenotyping, and genetic testing, this process is often constrained by incomplete family history, ambiguous presentations, limited access to genetic testing, and inconclusive genetic test results. Deep learning (DL) applied to fundus imaging presents a promising approach for automated inference of inheritance modes; however, development has been hindered by the low prevalence of IRDs and the scarcity of annotated datasets. In this study, we focus on retinitis pigmentosa (RP), a highly heterogeneous disorder in both clinical presentation and genetic etiology. We present a first-in-class deep learning approach that leverages Vision Transformer (ViT) models to distinguish autosomal from X-linked RP using color fundus photography. To overcome challenges posed by limited data, we introduce an innovative variational autoencoder–based data expansion strategy, which improves inheritance pattern classification based on color fundus photos from 0.67 AUC to 0.79 AUC. Our findings demonstrate the potential of deep learning to uncover subtle phenotypic differences linked to genetic inheritance and introduce a novel training data augmentation method to render deep learning accessible to rare diseases.

## Introduction

For rare inherited retinal diseases (IRDs), determining the mode of inheritance (i.e. autosomal versus X-linked inheritance) is crucial for providing accurate genetic counseling, guiding family planning, and predicting disease progression. While genetic testing is now routinely performed for many IRD patients, determining inheritance patterns through mutational analysis continues to present significant challenges (Britten-Jones et al., 2024; Xu et al., 2014). The most accessible and cost-effective option, whole-exome sequencing (WES), focuses on the protein-coding region, but coverage is limited and may miss disease-causing variants in non-coding regions (Burdick et al., 2020; Ross et al., 2020). Though more comprehensive, whole-genome sequencing (WGS) has other limitations, such as difficulty in detecting certain types of variants and higher costs (Marian, 2012). Furthermore, the diagnostic yield of next-generation sequencing for patients with RP ranges from 50–75% (Lynn et al., 2024; Consugar et al., 2015), and approximately half of non-syndromic RP patients are cases with no family history of disease, which complicates the determination of inheritance patterns (Jin et al., 2008). Although determining inheritance patterns through sequencing and family history remains difficult, research indicates that distinct genetic inheritance patterns may result in subtle morphological features that are unfortunately challenging to detect by the human eye alone (Currant et al., 2021; Ortin Vela et al., 2024).

Artificial intelligence (AI) enhanced imaging tools may offer a direct, non-invasive alternative. In clinical medicine, contemporary deep learning (DL) models have achieved disease diagnostic accuracies comparable to those of experienced physicians (Zhou et al., 2023; Men et al., 2023; Hwang et al., 2023). More remarkable, some DL models can further discern sub-visual features—such as inferring biological sex or age from fundus photographs—that elude human observers (Berk et al., 2023; Korot et al., 2021; Nusinovici et al., 2022). This success, however, has come at the cost of prodigious data demands. In response, the field is converging on foundation-model strategies—both general and task-specific—that couple resource efficiency with strong cross-task generalization (Gani et al., 2020). General-purpose vision encoders typically require 142–300 million heterogeneous images to attain competitive performance (Oquab et al., 2023; Dosovitskiy et al., 2020), whereas task-tailored variants achieve state-of-the-art (SOTA) accuracy with 1.6–3.4 million curated ophthalmic images (Zhou et al., 2023; Shi et al., 2024; Qiu et al., 2024). Transfer learning techniques can further shrink data requirements by orders of magnitude to 70,000 to 100,000 images (Yang et al., 2024; Cohen et al., 2025). While these AI techniques have empowered detection and grading of common retinal conditions (Silva-Rodriguez et al., 2024; Sevgi et al., 2024), such as diabetic retinopathy (DR) (Men et al., 2023) and age-related macular degeneration (AMD) (Du et al., 2024), reduced data thresholds still pose a prohibitive barrier for fields like IRDs, where annotated datasets seldom exceed a few hundred cases owing to low prevalence and fragmented data stewardship (Decherchi, 2021).

One promising strategy for curbing data demands is to leverage generative AI, which can augment existing datasets with high-fidelity synthetic images. (Chaurasia et al., 2024; Chen et al., 2024; Kumar et al., 2022). Synthetic data has been generated and utilized in multiple settings that are challenged by scarce datasets, including the development of multiracial facial recognition models and the curation of customized organ models for surgical simulation and training (Park et al., 2024; Kimura et al., 2025). While showing great potential, many generative AI methods, particularly diffusion-based models, are prone to hallucination, where the produced outputs are too far detached from reality, creating synthetic images that are plausible but nonsensical (Rajpurkar et al., 2018). To address this, we explored the use of variational autoencoder (VAE). VAE is a generative model that learns to encode input data into a latent space defined by a probability distribution, typically a multi-variate Gaussian. During training, the model optimizes a loss function that balances reconstruction accuracy with regularization, ensuring the latent space conforms to a known prior distribution. To generate synthetic data, new samples are drawn from this prior distribution and passed through the decoder network to produce novel but statistically consistent outputs (Wei & Mahmood, 2021). By generating outputs that adhere to specific input data, VAE is a more controllable generative model than Diffusion, mitigating the problem of hallucination. Previously, we demonstrated that VAE-enhanced synthetic datasets significantly improved glaucoma detection by ViT (Chen et al., 2024). Here we report the development of a second-generation VAE-based data enhancement workflow to deliver dataset diversity beyond previous methods and explore how this new functionality can enable access of DL methods to rare diseases.

## Methods

### Patient Cohort

Fundus photographs were acquired from patients seen at University of California San Francisco between October 2018 and August 2024 with a familial and/or sequencing-confirmed diagnosis of non-syndromic retinitis pigmentosa, including autosomal dominant (AD), autosomal recessive (AR), X-linked recessive (XR), or patients with sequencing-confirmed X-linked carrier (XLC) status. A total of 132 color fundus photographs were included in the full dataset. Symptom duration was determined from chart review by a retinal specialist with IRD expertise (J.L.D.), and was defined as the length of time between patient-reported onset of visual symptoms and imaging date, rounded up to the nearest year. Asymptomatic patients were assigned a symptom duration of 0 years. Statistical analysis was performed with GraphPad Prism software (10.0.3). The study adhered to the tenets of the Declaration of Helsinki and was approved by the UCSF Institutional Review Board, which determined that this retrospective study qualified for a waiver of informed consent.

### Data Preprocessing

For the purpose of confirming laterality, only fundus photos with clearly visible macula and optic nerve head (ONH) structures were included. If the patient had multiple imaging dates, only the most recent date was included for review. Fundus photos from the most recent date were manually assessed for image quality (excessive blur, artifact, sufficient field of view). In addition, all right eye (OD) images were horizontally flipped to match the image orientation of the left eye (OS) prior to model training. Eyes without images meeting these quality criteria were excluded.

### Autoencoder

To address the problem of overfitting caused by data scarcity, we employed a variational autoencoder framework to generate synthetic data. Two variations of autoencoder expansion were investigated: random noise expansion (Gen 1) and pair-wise combinatorial expansion (Gen 2).

#### I. Random Noise Expansion (Gen 1) VAE

Details of this first-generation expansion and training procedure were previously published (Chen et al., 2024). Briefly, synthetic images were generated by introducing noise into the latent space using four noise distributions: constant, Gaussian, uniform, and sinusoidal. For each image, one strategy was randomly selected and applied with a randomly sampled strength parameter from .05 to 1 to the embedding, leading to additional variations in the output images. Each image along with their random noise expansion was added to the training set resulting in a two-fold expansion of the training data. Our VAE utilized a dual-level ResNet-based encoder-decoder structure trained on ImageNet with pixel-wise reconstruction loss.

#### II. Pairwise Combinatorial Expansion (Gen 2) VAE

**Figure 1:**
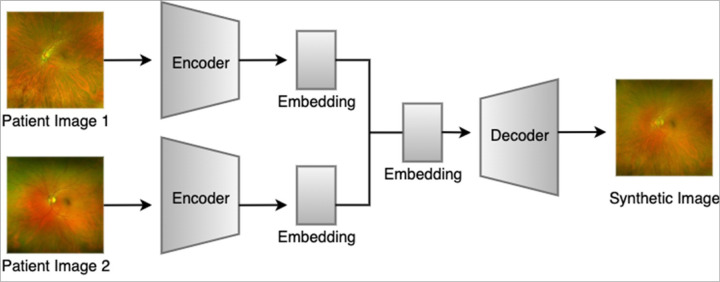
Two-way combinatorial synthetic image generation using variational autoencoder.

**Figure 2:**
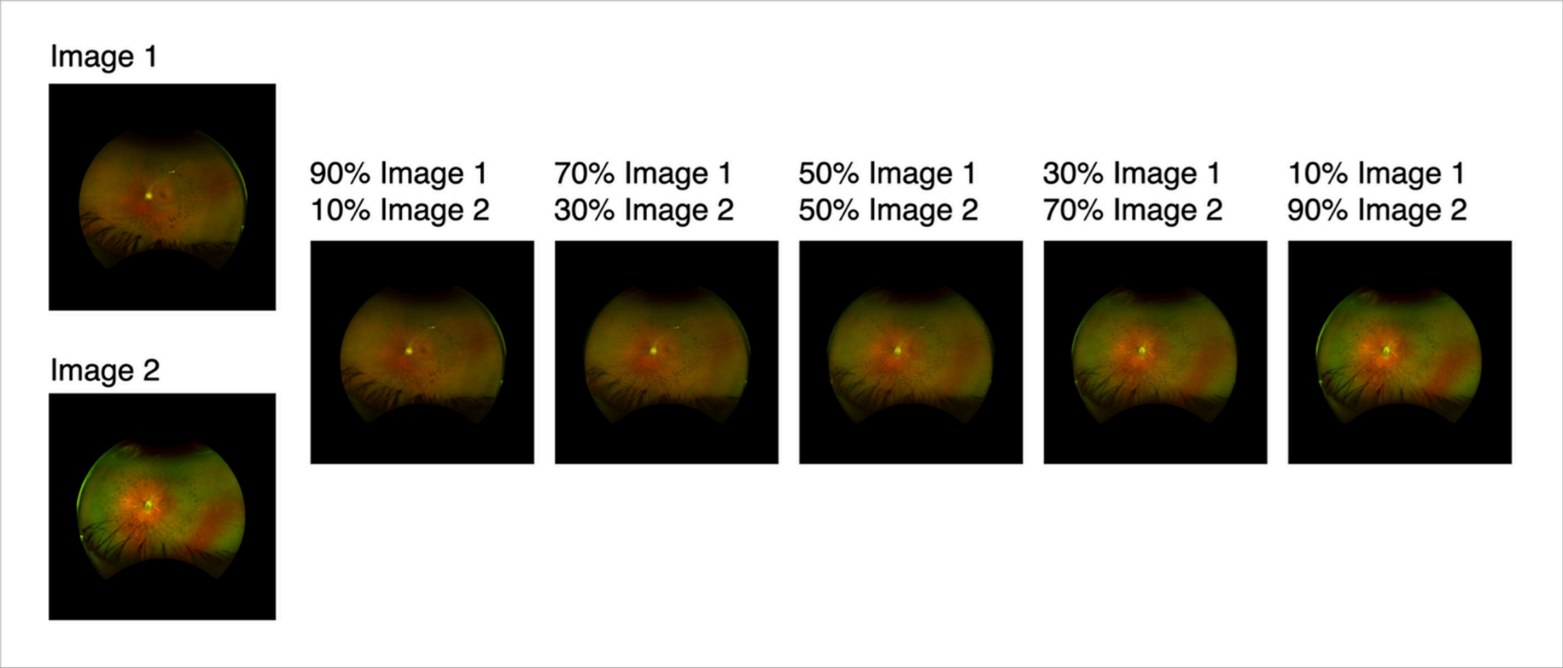
Examples of Synthetic Images based on Autosomal Dominant (AD) RP images. Left to right A/B ratios: 0.1, 0.3, 0.5, 0.7, 0.9.

To expand data enhancement capabilities in terms of both quantity and diversity, we developed a 2nd generation framework to perform pairwise combinatorial expansions using VAE. Here, synthetic images were generated by combining the latent representations of every possible pair of training images that share the same genotype label (Fig. 1). This pairwise structure ensures that every unique two-image combination within a genotype class contributes to the synthetic dataset. For each image pair, we encoded both images using a pretrained variational autoencoder (AutoencoderKL) and linearly combined their latent vectors using a predefined set of mixing ratios of 0.1, 0.3, 0.5, 0.7, and 0.9, as illustrated in Fig. 2. Each mixing ratio determines the relative weight between the original image and its paired image in the latent space. This process is repeated for n x n combinations for each label, where n is the number of images with the same label, augmenting each inheritance mode by C (n, 2) images. The resulting composite latent vector is then decoded to produce a synthetic image.

### Vision Transformer Training and Evaluation

**Figure 3:**
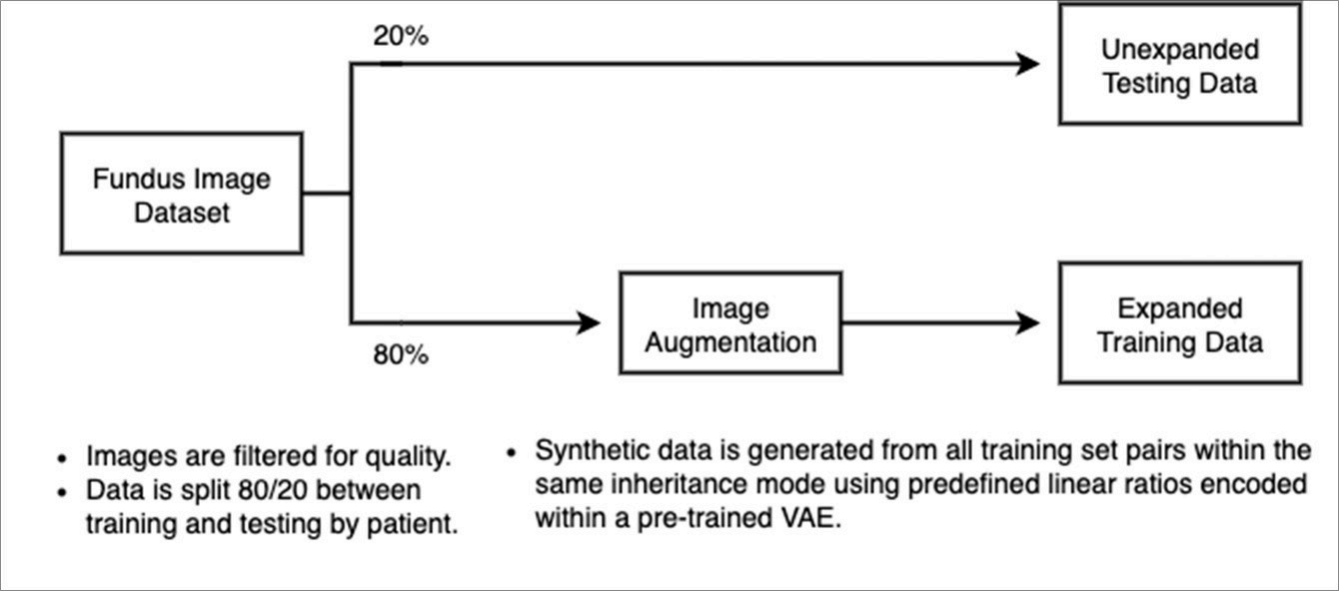
Workflow for Data Expansion and Vision Transformer Model Training. VAE = Variational Autoencoder, ViT = Vision Transformer.

The pretrained foundation model used in this study (Google’s vit-base-patch16-224-in21k) was initialized with pretrained weights from ImageNet-21k and modified for binary classification. We applied an 80/20 train-test data split, ensuring that the fundus images of both eyes from the same patient are incorporated into the same split, and trained two ViT models: one using only the original fundus images and the other incorporating both original and synthetic images (Fig. 3). To prepare the input images for training and evaluation, all images were resized to 224×224 pixels, matching the input size expected by the ViT model. Resizing images to a resolution of 224×224 pixels is a standardized preprocessing step in Vision Transformer (ViT) model, as demonstrated in multiple ViT-based retinal imaging studies (Wang et al., 2024; Powroznik et al., 2025). Training was performed for 30 epochs, using the AdamW optimizer with a learning rate of 5e-05. During training, we applied data augmentations using a randomized resizing crop followed by a randomized horizontal flip, introducing variability and reducing overfitting. Cross validation was performed by re-sampling to generate representative train/test splits. Synthetic images were generated from and added to only the training sets. To calculate mean accuracy, recall, and specificity, model performances were averaged over five-fold cross-validation. Pooled AUCs were calculated by aggregating labels and predictions generated across all validation sets.

## Results

### Retinitis pigmentosa (RP) patient demographics

**Figure 4:**
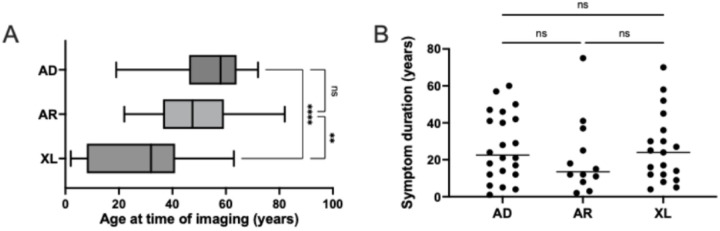
Patients mean age (panel A) and median symptom duration at time of imaging (panel B). AD = Autosomal Dominant, AR = Autosomal Recessive, XL = X-linked Recessive and X-linked Carrier (combined for anonymization purposes). ** denotes p-value < 0.005 and *** denotes p-value < 0.0001

Our final cohort included 105 eyes from 53 retinitis pigmentosa (RP) patients, for a total of 132 wide-field color fundus photos (Table 1). X-linked recessive (XR) and X-linked carrier (XLC) patient characteristics were reported as a single category to protect confidentiality. Patients’ mean age at time of imaging was 53 years (± 16 years) for autosomal dominant (AD), 49 years (±17 years) for autosomal recessive (AR), and 26 years (± 19 years) for XR or XLC (Table 1, Figure 4). Mean patient ages were significantly different (ordinary one-way ANOVA with Tukey’s multiple comparisons test, p-value < 0.01) between the autosomal (AD, AR) and X-linked (XL) groups, in line with known earlier onset of symptoms in X-linked RP (De Silva et al., 2021). Patient median symptom duration at time of imaging was 27 years (± 18 years) for AD, 22 years (± 21 years) for AR and 26 years (± 19 years) for XL, with no significant inter-group differences by one-way ANOVA (Table 1, Figure 4).

### Vision Transformer classification of disease inheritance mode in RP

**Figure 5:**
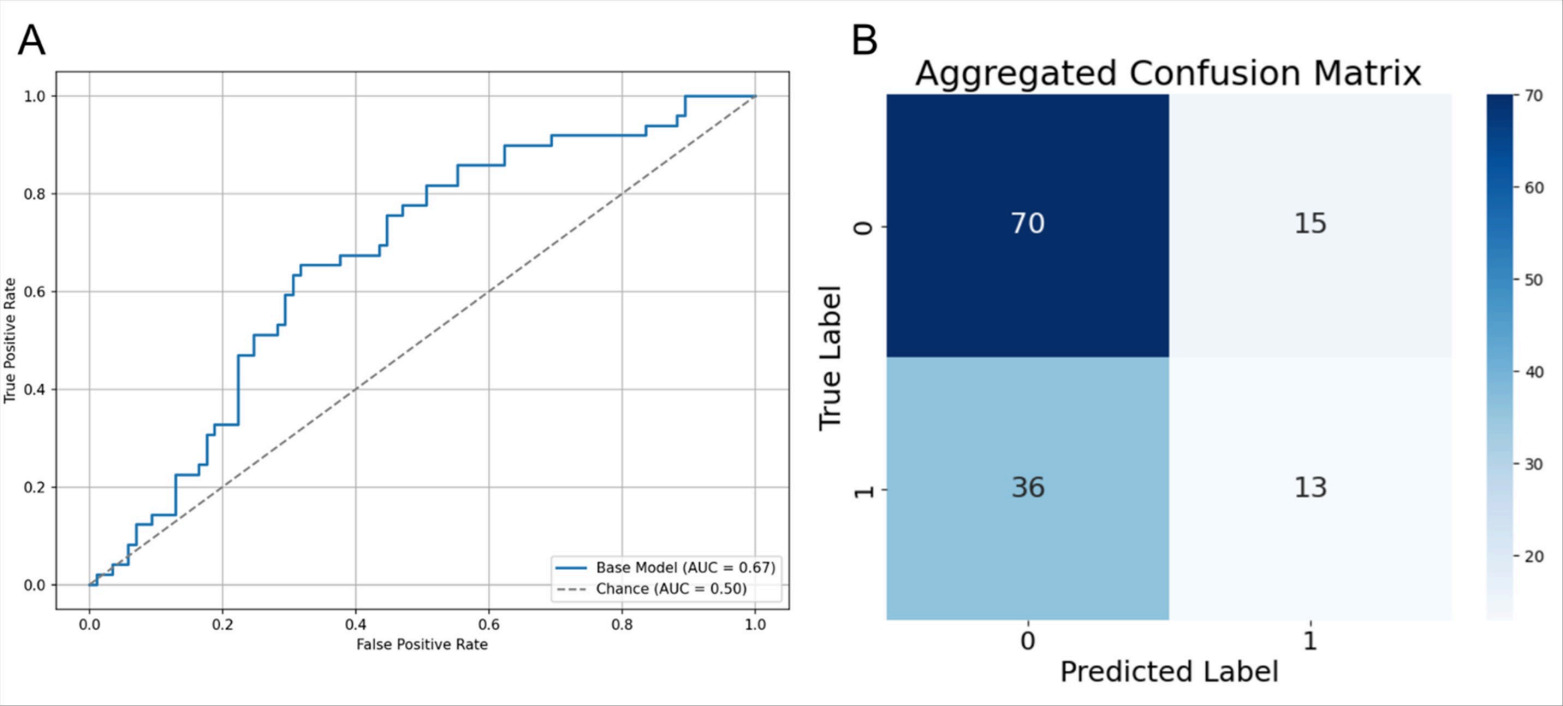
A. Receiver Operating Characteristic (ROC) curve with pooled AUC reported for RP ViT base (unexpanded) model. B. Confusion matrix for the base model.

We first evaluated our retinitis pigmentosa inheritance Vision Transformer (RP-ViT) base model trained on color fundus photo dataset without synthetic training data enhancements. For the purpose of binary model classification, we combined the four inheritance modes into two classes, autosomal (AR and AD) and X-linked (XR and XLC). For the RP-ViT base model, pooled AUC was 0.67, mean accuracy was 0.62 ± 0.05, and mean specificity was 0.55 ± 0.07 (Figure 5, Table 2).

### Autoencoder Enhancement of ViT-based classification of RP inheritance mode

#### I. Random Noise Expansion (Gen 1)

**Figure 6:**
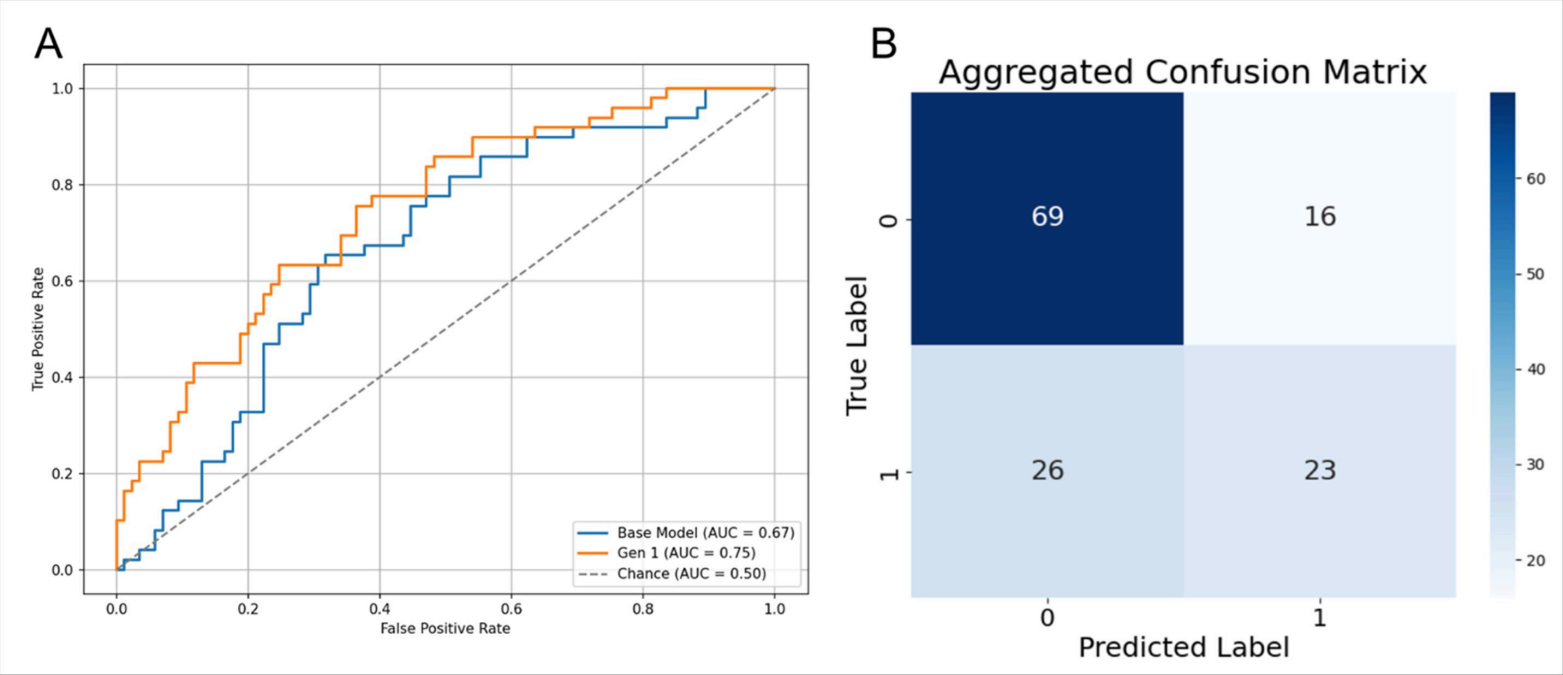
A. Receiver Operating Characteristic (ROC) curves for RP-ViT base and Gen 1 (random noise) expanded models. B. Confusion matrix for Gen 1 (random noise)-expanded models.

We trained and evaluated RP-ViT using a two-fold augmented dataset with synthetic images produced by the first-generation VAE method (Chen et al., 2024). This improved pooled AUC to 0.75, mean accuracy to 0.69 ± 0.05, and mean specificity to 0.64 ± 0.07 (Figure 6, Table 2).

#### II. Pair-wise Combinatorial Expansion (Gen 2)

**Figure 7:**
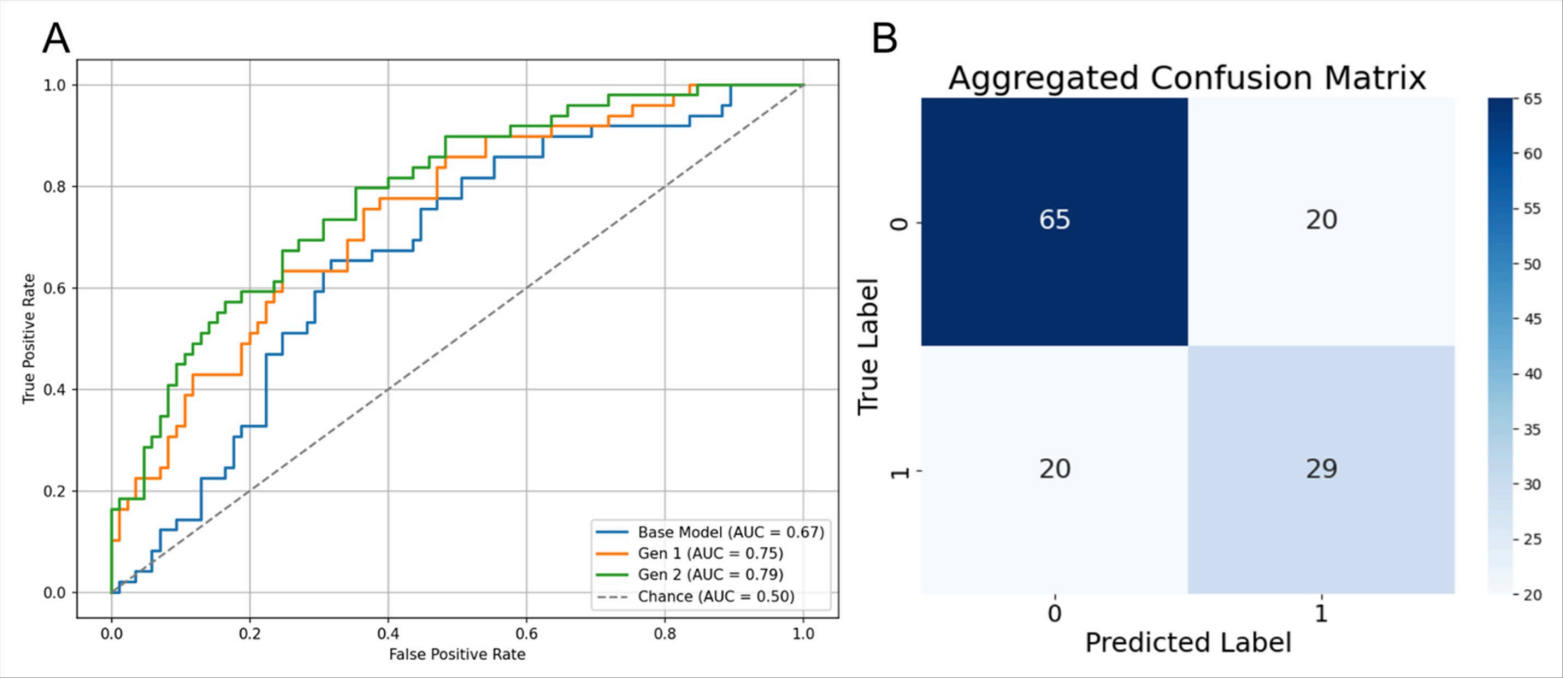
Receiver Operating Characteristic (ROC) curves for RP-ViT base, Gen 1 random noise-expanded, and Gen 2 pair-wise combinatorial expanded models. B. Confusion matrix for Gen 2 RP-ViT model.

Finally, we evaluated RP-ViT trained on a combinatorial pair-wise expanded dataset with synthetic images produced by our second-generation VAE method. This final model outperformed both the base model and the random noise-expansion RP-ViT models on all measured metrics with a pooled AUC of 0.79, mean accuracy of 0.71 ± 0.10, and mean specificity of 0.68 ± 0.10 (Figure 7, Table 2).

## Discussion

Deep-learning systems have achieved near-expert performance in detecting prevalent retinal disorders—most notably diabetic retinopathy and age-related macular degeneration—powered by the vast imaging datasets generated through population-wide screening initiatives (Aggarwal et al., 2021; Cen et al., 2021; Shoaib et al., 2025). Yet their translation into subspecialty clinics has been limited, with few algorithms undergoing rigorous, real-world validation in expert settings where diagnostic subtleties matter most (Abràmoff et al., 2018; Li et al., 2023). The gap widens further for rare inherited disorders such as retinitis pigmentosa, where patient numbers are small and existing datasets fall several orders of magnitude below typical deep-learning requirements. It thus remains an open question for the field whether AI models trained on such limited cohorts can deliver actionable insights.

In this study, we report on the application of generative AI and deep learning to classify genetic inheritance patterns in rare retinal diseases. Specifically, we demonstrate the feasibility of using a state-of-the-art (SOTA) vision-based deep learning model (ViT) to identify modes of inheritance from retinitis pigmentosa (RP) fundus images. To enable application of ViT on an inherently scarce dataset that is orders of magnitude smaller than previously reported deep learning usage cases, we developed novel methods of training data enhancement. Using a variational autoencoder, we introduced both random-noise (Gen 1) and pair-wise combinatorial (Gen 2) expansions of training data. Results show that while both methods can improve ViT performance, Gen 2 (which fuses pairs of labeled images to simulate biologically plausible variants) offers greater range of quantity and diversity in its synthetic data, leading to improvements beyond Gen 1 methods. By generating synthetic data via the fusion of real patient images rather than using diffusion-based methods, we minimize hallucination and maximize clinical fidelity and relevance of the resultant synthetic training data.

Our findings introduce two important concepts in clinical AI methodology: first, the ability to identify hidden patterns that correlate with inheritance mode and are invisible to human detection, and second, an encoder-decoder generative AI approach to alleviate scarce-data limitations of rare diseases. To our knowledge, this is the first report to use deep learning to classify images by mode of inheritance in retinitis pigmentosa (RP). We chose a binary classification approach based on both clinical and model design considerations. Classification of inheritance patterns is an important first step for diagnosis and for selecting appropriate genetic testing approaches and interpretation, which may inform the patient about relevant clinical trials or treatment options (Ullah et al., 2025; Lam et al., 2021; Gocuk et al., 2023). Distinguishing between X-linked vs. autosomal inheritance for rare IRDs can improve genetic counselling and family planning, and also enable more targeted diagnostic testing approaches, potentially improving the yield of diagnostic testing. In addition to its clinical relevance, our binary classifier model demonstrated better accuracy compared to a multi-class classifier. We suspect this difference may be due to the inherent limitations of classifiers in handling multiclass problems, as well as the small size of rare disease datasets (Allwein et al., 2000). Multiclass classifiers are intrinsically more data-hungry, a limitation that becomes pronounced in rare-disease settings where sample counts are low. While our data augmentation pipelines aim to address this data scarcity problem, their benefits, particularly that of Gen 2, scale with the size of the native dataset because each new image arises from two distinct originals. Consequently, the smallest cohorts (e.g. <20 images) still pose a challenge. Ongoing efforts therefore aim to further advance data expansion techniques to enable AI tools that perform robustly even with very small datasets, thereby improving classification accuracy in multi-class problems.

Beyond building a more robust data-enhancement architecture, it is our hope that a discovery-focused approach to AI tool development may uncover previously unrecognized deep biological signatures. Pathology unfolds across many measurement axes (e.g. structural, functional, and molecular), each contributing a slice of a high-dimensional landscape. Integrating complementary modalities, such as optical-coherence tomography, fundus imaging, and electrophysiology, provides a more complete representation of inherited retinal disorders and, accordingly, may improve DL classification performance (Hwang et al., 2025).

We acknowledge several limitations to our approach. First, we relied on a limited dataset from a single institution. While UCSF is a tertiary referral center and attracts patients worldwide, our cohort for this initial report is nevertheless limited. Our current work serves as a proof-of-concept, laying groundwork for promoting the development of future multi-center studies to validate the reported framework across more diverse populations.

Second, the autosomal and X-linked groups in our cohort differed significantly in mean age—an expected reflection of the earlier onset typical of X-linked IRDs. Although this age disparity may serve as a confounding factor, it also represents a genuine clinical distinction in onset and progression between inheritance patterns; the differences we observed likely reflect differences in disease severity that correlate with inheritance pattern. Third, similar to other deep learning models, our method involves a level of complexity that can obscure the interpretability of its decision-making process —making it difficult to determine which features were most relevant to the model predictions. While established interpretability tools exist for earlier architectures such as CNNs, the self-attention mechanisms used in Vision Transformers (ViTs) make it more challenging to generate intuitive or clinically recognizable explanations (Doncevic & Herrmann, 2023). To address this limitation, there is ongoing work to develop a multimodal large language–vision model to articulate its visual reasoning for medical image analyses.

In conclusion, this study highlights the potential of deep learning to contribute to the diagnostic workflow for inherited retinal diseases. By integrating VAE-powered data enhancement with ViT-driven binary classification, we report a novel and effective framework for mitigating the limitations imposed on AI tool development by inherently scarce data and may provide information about disease severity in resource-limited settings where genetic testing is not available or results are indeterminate. The reported framework enabled classification of RP inheritance mode using a small dataset, fulfilling an important clinical objective and underscoring the potential of AI to enhance healthcare for all patient populations, irrespective of disease prevalence.

## Figures and Tables

**Table 1.** Study cohort. AD = Autosomal Dominant, AR = Autosomal Recessive, XL = X-linked Recessive and X-linked Carrier (combined for anonymization purposes).

	AD	AR	XL	All
Patients	22	13	19	54
Sex				
Males	11	9	12	32
Females	11	4	7	22
Median symptom duration in years (SD)	27 (18)	22 (21)	26 (19)	
Mean age at time of imaging in years (SD)	53 (16)	49 (17)	26 (19)	

**Table 2.** Performance statistics for vision transformer (ViT) RP classification models, with base (unexpanded) and synthetically enhanced (Gen 1, Gen 2) datasets.

Expansion	AUC	Pooled AUC^1^	Accuracy	Specificity
Base	0.70 *(0.64, 0.76)*	0.67	0.62 *(0.57, 0.67)*	0.55 *(0.48, 0.62)*
Random (Gen 1)	0.76 *(0.68, 0.84)*	0.75	0.69 *(0.64, 0.74)*	0.64 *(0.57, 0.71)*
Pair-wise (Gen 2)	0.79 *(0.68, 0.90)*	0.79	0.71 *(0.61, 0.81)*	0.68 *(0.58, 0.78)*

1 Pooled AUC refers to AUC calculated from combined classifications across all folds.

## Data Availability

The datasets used and/or analyzed during the current study available from the corresponding author on reasonable request.
